# Low Empathy in Deaf and Hard of Hearing (Pre)Adolescents Compared to Normal Hearing Controls

**DOI:** 10.1371/journal.pone.0124102

**Published:** 2015-04-23

**Authors:** Anouk P. Netten, Carolien Rieffe, Stephanie C. P. M. Theunissen, Wim Soede, Evelien Dirks, Jeroen J. Briaire, Johan H. M. Frijns

**Affiliations:** 1 Department of Otorhinolaryngology and Head & Neck Surgery, Leiden University Medical Center, Leiden, The Netherlands; 2 Department of Developmental Psychology, Leiden University, Leiden, The Netherlands; 3 Dutch Foundation for the Deaf and Hard of Hearing Child, Amsterdam, The Netherlands; 4 Leiden Institute for Brain and Cognition, Leiden, The Netherlands; University of Vienna, AUSTRIA

## Abstract

**Objective:**

The purpose of this study was to examine the level of empathy in deaf and hard of hearing (pre)adolescents compared to normal hearing controls and to define the influence of language and various hearing loss characteristics on the development of empathy.

**Methods:**

The study group (mean age 11.9 years) consisted of 122 deaf and hard of hearing children (52 children with cochlear implants and 70 children with conventional hearing aids) and 162 normal hearing children. The two groups were compared using self-reports, a parent-report and observation tasks to rate the children’s level of empathy, their attendance to others’ emotions, emotion recognition, and supportive behavior.

**Results:**

Deaf and hard of hearing children reported lower levels of cognitive empathy and prosocial motivation than normal hearing children, regardless of their type of hearing device. The level of emotion recognition was equal in both groups. During observations, deaf and hard of hearing children showed more attention to the emotion evoking events but less supportive behavior compared to their normal hearing peers. Deaf and hard of hearing children attending mainstream education or using oral language show higher levels of cognitive empathy and prosocial motivation than deaf and hard of hearing children who use sign (supported) language or attend special education. However, they are still outperformed by normal hearing children.

**Conclusions:**

Deaf and hard of hearing children, especially those in special education, show lower levels of empathy than normal hearing children, which can have consequences for initiating and maintaining relationships.

## Introduction

Hearing impairment poses many challenges to the developing child. Deaf and hard of hearing (DHH) children for instance frequently encounter language and communication problems. These difficulties in communication may result in reduced opportunities for incidental learning. Especially abstract concepts such as emotions are therefore more difficult to understand for children with hearing loss [1]. Regulating and understanding one’s own emotions is essential for the development of adequate empathic abilities. Consequently, DHH children are prone to develop lower empathic skills than normal hearing (NH) peers. Because empathy is of major importance in initiating and maintaining social relationships, this could have ongoing consequences in the development of DHH children.

### Empathy

Empathy is defined as the ability to perceive and understand another person’s emotional state and the competence to appropriately respond to others’ emotions [2,3]. It is needed to induce prosocial behavior: free-willing behavior to benefit others [4]. Therefore, empathy is often referred to as ‘social glue’ in relationships [4–6].

From a developmental perspective, empathy has been divided into different layers: affective empathy, cognitive empathy and prosocial motivation. Affective empathy, also known as emotional contagion, is the process in which the emotional states of others cause a level of arousal in the observer. It consists of non-conscious behavioral mimicry of others’ facial, vocal, and bodily expressions [7]. This ‘mirroring of emotions’ is thought to be present at birth and originates from the Mirror Neuron System (MNS) in the brain. Through functional Magnetic Resonance Imaging (fMRI) studies, neuroscientific research has shown these neural networks. For instance, making a sad face or observing a sad face both activate the MNS via the amygdala and the anterior insula of the brain. This motor activation is then associated with an emotion representation; the person acknowledges a sad feeling [8–10]. These patterns strongly suggest that the formation of cortical representations about one’s own feelings is a necessary condition to engage in vicarious predictions about the emotions of others [5] (see Lamm & Majdandžić for an in-depth discussion of the plausibility of this assumption [11].

Whereas young children become upset and need comforting themselves through affective empathy, also referred to as ‘contagious crying’ or ‘emotional sharing’, around the age of two children change from a self-focused perspective towards another-focused perspective. Consequently, children gradually become to understand that their sad feelings are caused by another person in distress. This evokes an urge to support or comfort that person, as to relief their distress [3,5,12].

Cognitive empathy develops as children grow older and involves a more sophisticated comprehension of the other person’s emotional state [13]. The child starts to understand why the other is upset. Understanding emotions in others serves different goals. First, the observer is capable to distinguish between its own and the other’s emotions and thereby decreasing their own feelings of distress. Second, understanding the other leads to an increased tendency to support the other, and to care for the other [11]. This intrinsic prosocial motivation is essential because it signals to the person in distress that the observer pays attention to and highly values their emotions. He or she understands what is happening and wants to support. Moreover, a stronger level of cognitive empathy can also help to overcome in-group preferences [11]. The long-term purpose of cognitive empathy and prosocial motivation is to induce and maintain good social relationships [12]. As such, the development of cognitive empathy is largely dependent on social learning. fMRI studies have indirectly shown this as the relation between the MNS and self-reported cognitive empathy is less clear than for affective empathy [7,11]. For social learning to develop, this requires incidental learning skills; unplanned and unorganized learning abilities, with no educational intentions. Social learning takes place while interacting with others, and by trial-and-error.

A lack of empathy is associated with violence, aggression, criminality, and insensitive and unemotional behavior [14]. Empathic dysfunction has been associated with several psychiatric disorders such as psychopathy, autism spectrum disorders [15], conduct disorder, acquired sociopathy [16], and schizophrenia [17]. Children and adolescents who show little or no empathy are deemed to fail in our social world, and are put aside as having antisocial behavior. These behavioral problems may lead to the development of an antisocial personality disorder later in life [18]. Hence, it is of major importance for children to adequately develop empathic skills.

### Empathy in deaf and hard of hearing children

Little is known about the development of empathy in DHH children. However, certain prerequisites for successful empathic maturation, such as emotion recognition and regulation together with development of a Theory of Mind (ToM), have recently been addressed in this population. Studies show lower levels of emotion recognition and labelling of emotions in deaf preadolescents than in NH peers. In this population the onset of deafness was related to the ability to recognize emotions. Prelingually deaf preadolescents were more vulnerable than their postlingually deaf peers [19]. Regarding emotion awareness, DHH children were found to be less able to address multiple emotions in the negative domain simultaneously (e.g., anger and sadness) than NH peers during several emotion tasks. In the same study, children had to focus on approaching strategies towards an emotion-evoking situation. The results show less effective emotion regulation in DHH children than in NH peers [20]. ToM has been measured in toddlers with cochlear implants (CIs). Initially children with CIs were found to perform as well as NH children. However, at an older age they fell behind on more advanced ToM abilities such as false belief tasks [21]. Regarding their empathic behavior, no differences were found between young children with a CI and NH peers [5]. Yet, because of the young age of these children (1–5 years), only the affective domain of empathy could be taken into account in this study.

### Present study

Because of the continuous development of cognitive empathy in childhood and preadolescence we are interested whether empathic abilities in DHH children and adolescents differ from those of their NH peers. Therefore, the aim of this study was to determine the differences in the levels of self-reported and observed empathy between DHH on the one hand, and NH children and adolescents on the other. To identify those factors that may be most influential for the levels of empathy in DHH children we also investigated the influence of several audiological factors on empathic abilities, such as language development, intelligence, degree of hearing loss, age at intervention of hearing loss, type of device, mode of communication, and educational setting.

On the basis of the research mentioned above we expected to find equal levels of affective empathy in DHH and NH children. However, regarding the development of cognitive empathy and prosocial motivation we expected DHH children to fall behind as a consequence of, among other things, their impaired ToM development. Concerning several audiological variables such as type of hearing amplification, it has been reported that DHH children wearing CIs experience lower levels of behavioral problems than children wearing Hearing Aids (HAs) [22]. Therefore, we expected to find differences in empathic ability between these two groups.

Gender differences have been described frequently in the literature. Girls consistently report higher levels of affective empathy and prosocial behavior than boys. Some researchers doubt these conclusions. They hypothesize that the reported differences are a result of differences in social desirability between boys and girls [8,23]. If true, we would find higher levels of self-reported affective empathy and prosocial motivation in girls, regardless of their hearing status but equal levels of empathy and supportive behavior during observations.

Due to the improved developmental outcomes after early intervention programs as reported by Yoshinaga-Itano et al. [24], we expected a relation between age at detection and intervention of hearing loss, and empathic abilities. Educational placement (mainstream or special schools) and mode of communication (spoken or sign language) have been reported to be related to levels of psychopathology in DHH children [25–28]. We therefore expected that children attending mainstream education and using spoken language as their preferred mode of communication show higher levels of empathy.

## Material and Methods

### Participants

We recruited 122 DHH children and a control group consisting of 162 NH children from all over The Netherlands and the Dutch-speaking part of Belgium to participate in this study. All children were between 9 and 16 years of age at time of assessment. The age of 9 as a cut-off point was chosen because the children needed to be able to reflect on their own emotions and behavior [29]. All children had an IQ of 80 or higher and no other known disabilities besides their hearing loss. Of all DHH children, 52 were fitted with a CI and 70 children wore conventional HAs. Hearing impairment was defined as experiencing a loss of ≥40 dB in the best ear that was detected pre- or perilingually. Children with postlingual onset or detection of hearing loss were excluded. The NH group was matched with the DHH group on sex and mean age. As can be seen in Table 1, gender, intelligence, socio-economic status (SES), and age did not differ between the groups. No differences were found in type of school and mode of communication when comparing children wearing a CI with children using HAs. The onset of hearing impairment differed between the two groups; χ^2^ (1, *n* = 115) = 3.92, *p*<.05. The HA-group presented more perilingual onset of hearing impairment than the CI group. As expected the degree of hearing loss differed between the two groups χ^2^ (2, *n* = 114) = 73.62, *p*<.001. Children with a CI mainly experienced profound losses whereas children with HAs showed more moderate to severe hearing losses. Permission for this study was granted by the Medical Ethics Committee of the Leiden University Medical Center under number P10.137.

**Table 1:** Demographic characteristics of participants.

	Total study population N = 284		HI study population N = 122	
	HI	Controls	CI	HA
No. of children	122	162	52	70
Age				
Mean—in years (SD)	11.9 (1.8)	11.9 (1.3)	11.8 (2.0)	12.0 (1.7)
Range—in months	100–194	99–176	100–194	110–188
Gender				
Male (%)	60 (49)	73 (45)	24 (46)	36 (51)
Socioeconomic Status (SD)	11.5 (2.3)	11.7 (2.3)	11.7 (2.3)	11.3 (2.4)
Nonverbal intelligence (SD)	10.3 (2.8)	10.7 (2.5)	10.0 (2.7)	10.5 (3)
Language Skills (SD)	6.5 (2.7)	7.0 (1.9)	6.1 (2.8)	6.7 (2.6)
Preferred mode of communication				
Oral language only (%)	94 (77)		39 (75)	55 (78)
Sign-supported Dutch (%)	26 (21)		13 (25)	13 (19)
Sign language only (%)	2 (2)		0 (0)	2 (3)
Type of education				
Regular education (%)	74 (61)	162 (100) *	32 (62)	42 (60)
Onset of hearing loss				
Prelingual (%)	103 (84)		48 (92)	55 (78) *
Perilingual (%)	12 (10)		2 (4)	10 (14) *
Unknown (%)	7 (6)		2 (4)	5 (7)
Degree of hearing loss				
Moderate—40–60 dB (%)	29 (24)		0 (0)	29 (41) **
Severe—61–90 dB (%)	25 (21)		1 (2)	24 (34) **
Profound—>90 dB (%)	60 (49)		49 (94)	11 (16) **
Unknown	8 (6)		2 (4)	6 (9)
Age at detection of hearing loss—in months (SD)	19.1 (15.7)		14.6 (10.4)	22.9 (18.3) **
Age at first hearing aid acquisition—in months (SD)	24.8 (17.0)		17.3 (10.2)	31.2 (18.9) **
CI characteristics				
Age at implantation (CI)—in months (SD)			44.5 (32.6)	
Duration of CI use—in months (SD)			99 (33)	
Bilateral CI (%)			13 (25)	

Abbreviations: HI Hearing Impaired, CI Cochlear Implant, HA Hearing Aid, SD Standard Deviation.

* *p*<.05,

** *p*<.01

### Procedure

To increase the external validity of our findings, we tried to ensure diversity in our study population and recruited children all over The Netherlands and the Dutch-speaking part of Belgium via hospitals, speech- and hearing centers, primary and secondary schools, and special schools for the deaf. Written parental informed consent was obtained for all participating children. The assessment was carried out in a quiet room. Before starting the tests, children were assured that their answers would remain anonymous. Questions appeared one by one on a laptop. Depending on their preferred mode of communication, DHH children could choose between two versions of the questionnaires: a written text, or a version in which this text was simultaneously accompanied by sign language. The questionnaires were assessed as part of a larger study on the socio-emotional development of DHH and NH children. In between several tests, the experimenter acted live emotions to observe empathic reactions and supportive behavior during the test session. Parents completed questionnaires at home, they were also asked to complete a list of background variables such as net income and level of education. A socioeconomic status (SES) score was calculated using the net income of the family, job and level of education of both parents. Audiological variables were extracted from the child’s medical and/or audiological notes.

### Materials

The instruments used in this study are described here. Psychometric characteristics of all questionnaires are shown in Table 2.

**Table 2:** Psychometric properties of empathy questionnaire, EAQ, EEQ and observations.

			Total study population N = 284				HI study population N = 122	
			HI	Controls	HI	Controls	CI	HA
	No. of items	Answer range	Crohnbach's alpha		M (SD)		M (SD)	
Self report								
Empathy Questionnaire	18	1–3	.83	.81	2.2 (0.3)	2.4 (0.3)**	2.2 (0.3)	2.3 (0.3)
*Affective empathy*	7		.66	.68	1.9 (0.4)	2.1 (0.4)	1.9 (0.4)	2.0 (0.4)
*Cognitive empathy*	5		.68	.66	2.3 (0.4)	2.5 (0.4)**	2.3 (0.4)	2.3 (0.4)
*Prosocial motivation*	6		.76	.72	2.6 (0.4)	2.7 (0.3)**	2.6 (0.4)	2.5 (0.4)
EAQ—Attendance to others' emotions	5	1–3	.64	.60	2.3 (0.4)	2.5 (0.4)**	2.2 (0.4)	2.3 (0.4)
Parent report								
EEQ—Emotion recognition	6	1–5	.78	.74	2.6 (0.3)	2.6 (0.3)	2.6 (0.3)	2.5 (0.3)
Experimenters observation								
*Attention to emotion*	8	1–3			2.5 (0.4)	2.2 (0.4)**	2.4 (0.5)	2.5 (0.4)
*Supportive behavior*	1	1–3			2.6 (0.6)	2.9 (0.4)**	2.6 (0.6)	2.6 (0.5)
WISC non-verbal intelligence˜	26	0–7			10.3 (2.8)	10.7 (2.5)	10.0 (2.7)	10.5 (3)
CELF-IV language development˜	35	0–1			6.5 (2.7)	7.0 (1.9)	6.1 (2.8)	6.7 (2.6)

Abbreviations: HI Hearing Impaired, CI Cochlear Implant, HA Hearing Aid, SD Standard Deviation, EAQ Emotion Awareness Questionnaire, EEQ Emotional Expressivity Questionnaire.

* *p*<.05,

** *p*<.01,

˜ normscores.

#### Self-reported empathy

The Empathy Questionnaire for Children and Adolescents (EmQue-CA) consists of a total number of 18 items, scored by children on a 3-point Likert scale (1 = not true, 2 = somewhat true and 3 = true). The items measure the different levels of empathy: affective empathy, cognitive empathy and the urge to support the other. The ‘affective empathy’ scale defines to what extent emotions in others cause isomorphic feelings in the observer (e.g., “*If a friend is sad*, *I also feel sad*”). The scale measuring cognitive empathy defines to what level children understand the emotions they observe in others (e.g., “*When a friend is angry*, *I tend to know why”)*. The third scale prosocial motivation’ defines the tendency to support a distressed other (e.g., “*If a friend is sad*, *I like to comfort him*”). The Questionnaire was validated for NH children of 9 years and older [30,31]. The internal consistency of the scales is acceptable to good; and the questionnaire shows a good three-factor structure [30], which warrants that the questionnaire is suitable to make group comparisons [11].

From the Emotion Awareness Questionnaire (EAQ), the ‘attendance to others’ emotions’ scale was used (e.g., *If a friend is upset*, *I try to understand why*). Children rated how valuable they found other children’s emotions on a 3-point Likert scale (1 = not true, 2 = sometimes true, 3 = often true) [32]. The internal consistency of the scale is acceptable.

The ‘emotion recognition’ scale from the Emotion Expression Questionnaire (EEQ) was scored by parents (e.g., *Does your child know when you are angry*?). The questions were rated on a 5-point Likert scale (1 = (almost) never, 2 = rarely, 3 = sometimes, 4 = often, 5 = (almost) always) [2]. The internal consistency of the scales is good.

Measurement invariance was not assessed for the above described questionnaires. However, the questionnaires were specifically designed to use in different clinical groups (Children with specific language impairments, autism spectrum disorders, and DHH children). Therefore, items were formulated with short sentences to increase understanding. Previous studies have shown consistent and positive outcomes in these groups[30,31].

#### Observation of empathy

Participating children were faced with ‘live’ emotions from the experimenter to observe to what extent they would show empathic reactions. Multiple situations were acted out, which aimed to evoke attention for the situation and/or the experimenter’s emotion and prosocial responses directed at the experimenter. Before data collection started, experimenters were instructed on how to simulate emotions. Emotions were modeled by a psychologist experienced in simulating emotions for behavioral assessment purposes. Additionally, experimenters watched multiple video clips of emotion simulations, which were obtained during a pilot study. Specific instructions were provided regarding the duration and intensity of the emotions displayed, as well as regarding the verbal and non-verbal cues that accompanied these. Experimenters then practiced and video-recorded multiple emotion simulations themselves, and received feedback on their performance from the trainer. Training continued until all experimenters could simulate the emotions in a natural way, as judged by the trainer.

In the first situation, the experimenter pretended to receive text messages from a friend. The experimenter reached for her phone and pretended to read the first message, after which she shared with the participant that it contained an invitation from her friend to go to the movies that night. The experimenter had an excited, happy facial expression and said that she was looking forward to it. After that, she put away the phone and continued the test session. Approximately 30 minutes later the experimenter pretended to have received another text message. This time, she shared with the participant that her friend had to cancel the appointment, meanwhile showing a disappointed, sad facial expression. After five seconds, the experimenter stored her phone and carried on with the session. During and after revealing the second message, the experimenter observed the behavioral and verbal responses of the participant.

In the second situation, the experimenter pretended she could not find her pen. Earlier, the pen was placed outside the direct line of sight of the experimenter (i.e., behind a binder), but in full view of the participant. For a duration of ten seconds, the experimenter looked around and searched her bag, stating that she could not find her pen. Meanwhile, children’s responses were observed.

In the third situation, the experimenter collected testing materials and dropped one item on the floor. The experimenter looked at the item and said ‘oops’, but continued to gather the rest of the materials. Children’s behaviors in response to the situation were observed.

Children’s reactions across all three situations were scored on a checklist (1 = *no*, 2 = *slightly*, 3 = *yes*) and were grouped into ‘attention to emotions’ (e.g., looking at the experimenter) and ‘supportive behavior’ (e.g., returning the lost pencil). Unfortunately, due to time restraints scores from 9 CI children, 9 HA children and 1 NH child are missing.

#### Language skills and intelligence

Nonverbal intelligence of participants was assessed using two components of the Wechsler Intelligence Scale for Children-Third Edition (WISC): block design (duplicating geometric designs with cubes) and picture concepts (arranging pictures to create logical stories) [33]. These scores were compared with scores of earlier completed intelligence tests (either the Snijders-Oomen or the WISC) [34]. A high correlation was found previously by Theunissen et al. [22] making the shorter subtest a good reflection of the child’s intelligence level. The WISC has been proven to show excellent test-retest abilities and long-term stability[35].

Sufficient language abilities are regarded essential to ensure comprehension of the different questionnaires. This was tested using a sentence comprehension and a story comprehension task. Children using oral language as their preferred mode of communication completed the Dutch version of the Clinical Evaluation of Language Fundamentals—Fourth Edition (CELF-IV) [36,37]. The CELF has been proven to show high stability coefficients. Studies were conducted in several clinical groups including children with language disorder, and hearing impairment [10]. DHH children who preferred communicating by sign (supported) language completed subtests from the Assessment Instrument for Sign Language of The Netherlands [38].

### Statistical Analyses

Group demographics were compared using independent t-tests. To compare the levels of empathy (affective empathy, cognitive empathy and prosocial motivation) between the different subgroups repeated measures Multivariate Analysis of Variance (MANOVA) and Multivariate Analysis of Covariance (MANCOVA) were used. In case of differences between subgroups within the DHH children, when sample sizes were small (<40 children per group) the assumption of normality was violated. Therefore, to compare levels of empathy between these subgroups (e.g., uni- versus bilateral CI, pre- versus perilingual onset of hearing loss) a non-parametric test was chosen (i.e., Mann-Whitney U test). Correlations between the empathy subscales and audiological factors were calculated using Pearson’s correlations. These correlations were compared between the different groups using Fisher’s *r*-to-*z* transformations to be able to show significant differences between correlations. Statistical analyses were carried out using the program *SPSS* version 21.0 (IBM Corp., Armonk, NY).

## Results

### Self-reported empathy in DHH and NH children

To analyze the differences in self-reported empathy levels between children with a CI, those with HAs, and hearing children, we carried out a repeated measures MANOVA with Group (CI, HA, NH) as the between-subjects variable and self-reported empathy (affective empathy, cognitive empathy, prosocial motivation) as the within-subjects variable. The analysis showed a main effect for empathy (*F*_*HF*_ (1.97, 553.96) = 303.81, *p*<.001, *η*_*p*_^*2*^ = .52) and for group (*F* (2, 281) = 11.44, *p*<.001, *η*_*p*_^*2*^ = .08), which was qualified by an empathy x group interaction (*F*_*HF*_ (3.92, 553.96) = 2.46, *p*<.05, *η*_*p*_^*2*^ = .02). Post-hoc t-tests showed that on affective empathy children with CIs scored lower than the NH group. Scores on affective empathy by children with HAs did not differ from NH children. DHH children overall scored lower on cognitive empathy and prosocial motivation than NH peers, regardless of their type of hearing amplification.

**Fig 1:**
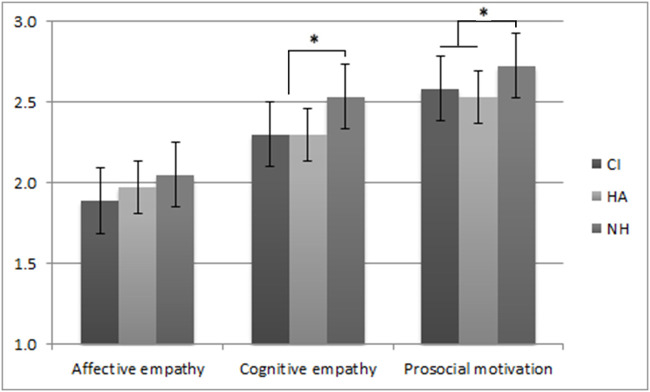
Mean empathy scores per group. * *p*<.01.

Because of the known influence of language development and intelligence on the socio-emotional development of DHH children, these variables were added as covariates in the analyses. In a MANCOVA that corrected for language development and intelligence, the main effect for group remained (*F* (2, 236) = 6.30, *p* = .002, *η*_*p*_^*2*^ = .05), but the interaction effect was no longer significant (*F*_*HF*_ (3.95, 465.57) = 1.55, *p* = .19, *η*_*p*_^*2*^ = .01). Language development was significantly related to the levels of empathy (*F*(1, 236) = 5.25, *p* = .02) whereas intelligence was not (Fig 1).

A gender x self-reported empathy repeated measures MANOVA was conducted to define differences in self-reported empathic abilities between boys and girls, regardless of their hearing status. Results showed a main effect for empathy (*F* (1.97, 555.75) = 393.96, *p*<.001, *η*_*p*_^*2*^ = .58) and for gender (*F* (1, 281) = 11.10, *p* = .001, *η*_*p*_^*2*^ = .04), which was qualified by an empathy x gender interaction (*F*_*HF*_ (1.97, 555.75) = 4.33, *p*<.05, *η*_*p*_^*2*^ = .02). Post-hoc analysis revealed that girls scored higher on affective empathy and prosocial motivation than boys. Equal levels of cognitive empathy were reported. The aforementioned results were combined in a 3 (hearing group) x 2 (gender) x 3 (self-reported empathy) repeated measured MANCOVA with language development and intelligence as covariates. The main effect for group remained (*F* (2, 233) = 5.75, *p* = .004, *η*_*p*_^*2*^ = .05) whereas the results no longer showed a main effect for gender (*F* (1, 233) = 3.54, *p* = .06, *η*_*p*_^*2*^ = .02).

Concerning attendance to others’ emotions, a 2 (DHH, NH) x 2 (boys, girls) one-way ANCOVA that corrected for language skills and intelligence revealed an effect for hearing group (*F* (1, 235) = 8.52, *p*<.01) and gender (*F* (2, 235) = 18.04, *p*<.001). NH children reported higher scores than DHH children and girls scored higher than boys. Language development was significantly related to the attendance towards others’ emotions (*F*(1, 240) = 4.80, *p*<.05). A one-way ANCOVA to compare the effect of hearing group and gender on emotion recognition as scored by parents corrected for language development and intelligence showed no differences between the hearing groups or gender (*F* (1, 182) = 0.03, *p* = .87 and (*F* (1, 182) = 0.065, *p* = .80, respectively).

### Observation of empathy and supportive behavior

Differences between gender and hearing status in observed empathic behavior during the live emotions tasks were assessed with language and intelligence as covariates. A 2 (DHH, NH) x 2 (boys, girls) mixed ANCOVA revealed an effect for hearing status and for gender; DHH children scored higher than their NH peers on emotion attention(*F* (1, 220) = 28.80, *p*<.001); regardless of their type of hearing amplification. Girls scored higher than boys (*F* (1, 220) = 10.94, *p* = .001). To compare DHH and NH boys and girls on their observed supportive behavior, a 2 (DHH, NH) x 2 (boys, girls) mixed ANCOVA was performed showing an effect for hearing status but not for gender (*F* (1, 220) = 16.03, *p*<.001 and *F* (1, 220) = .66, *p* = .42, respectively). Conversely to their ‘emotion attention’, NH children more often showed supportive behavior than DHH children.

### Audiological and socio-demographic factors influencing empathy

In order to properly examine levels of empathy between DHH children at special education (for the deaf and hard of hearing child) and at mainstream education, a MANCOVA was performed with school-type (special or mainstream) as the between-subjects variable, the self-reported levels of empathy as the within-subjects variables and language development as a covariate. The two groups did not differ in background and audiological characteristics (e.g., age at detection of hearing loss, age at intervention, intelligence, SES). The analysis showed a main effect for empathy (*F*_*HF*_ (2, 204) = 9.16, *p*<.001, *η*_*p*_^*2*^ = .08) and for school-type (*F* (1, 102) = 4.38, *p*<.05, *η*_*p*_^*2*^ = .04). Post-hoc ANCOVA’s revealed higher levels of cognitive empathy in DHH children attending mainstream schools than in DHH children attending special schools, (*F*_*HF*_ (1, 102) = 7.89, *p*<.01), whereas for affective empathy and prosocial motivation no significant differences were found (*F*_*HF*_ (1, 102) = 1.61, *p* = .21. and *F*_*HF*_ (1, 102) = .91, *p* = .34, respectively). No significant differences were found in observed empathic reactions nor in parent reported emotion recognition or attendance to others’ emotions comparing DHH children in mainstream and special education when corrected for their language skills.

When comparing the child’s preferred mode of communication DHH children using sign (supported) language scored lower on self-reported prosocial motivation and on observed attention to emotions than DHH children who preferred to use spoken language (*U* = 986.5, *z* = -2.95, *p* = .003 and *U* = 802.5, *z* = -2.32, *p* = .021, respectively). Two participants solely communicated by sign-language. All analyses were rerun without these two participants. The results did not differ.

No significant differences were found between the levels of empathy in children regarding the moment of detection of their hearing loss (i.e., pre- or perilingual). When comparing within the CI group, parents reported higher levels of emotion recognition in unilaterally implanted children compared to bilaterally implanted children (*U* = 82, *z* = -2.54 *p* = .01).

In the DHH group, the relation between several continuous audiological variables (degree of hearing loss, age at detection of hearing loss, age at intervention of hearing loss, age at implantation) and the levels of empathy (self-report, parent-report and observed) were analyzed by means of Pearson’s correlations. No relations were found between these variables and the different levels of empathy.

## Discussion

Empathy is an important capacity which helps to build and maintain positive social relationships [39]. It has been argued that affective empathy (i.e., feeling what the other person feels) is neurologically hard-wired, i.e., present in children despite their social learning experiences [35]. Yet, the level of cognitive empathy (i.e., understanding the other’s emotions) depends for instance on the extent to which children can participate in a social environment [39]. We hypothesized that DHH children would be seriously disadvantaged in this respect. The outcomes of this study support our hypothesis: DHH children report equal levels of affective empathy as NH peers. Even higher levels of attention to others’ emotions in DHH children than in NH children were found during an observation task. Yet, DHH children reported lower levels of cognitive empathy, and valued emotional information about other people as less important. Moreover, both a self-report and an observation task show less supportive behavior in the DHH group compared to NH peers. In other words, DHH children might feel what the other person feels, and also attend to those emotions, but they have less understanding of their causes; they value others’ emotions as less important, and also react less adaptively to supporting the person in distress. Yet, especially the capacity for cognitive empathy, whereby one is more inclined not only to feel for the other, but also take the perspective of the other person, is essential in overcoming in-group preferences and avoiding parochialism [11].

Consistent with other research in the domain of empathy, girls scored higher than boys on affective empathy and prosocial motivation. Only during the observation tasks, no differences were found between boys’ and girls’ tendency to behave supportive. Within the DHH group we see that children in mainstream schools, or those who used spoken language as their primary mode for communication, did better on cognitive empathy than their DHH peers in special schools or using sign or sign(-supported) language, respectively. Unfortunately, they are still outperformed on these abilities by NH children.

Although the level of affective empathy was equal in both groups, this was only after we controlled for the children’s language capacity. Language abilities were taken into account since previous studies have shown communication skills and interaction with others are improved by sufficient language skills, resulting in better socio-emotional development and fewer symptoms of psychopathology in DHH children [6,22,27,40]. However, impaired language skills only partly explain the lower empathic abilities we found in DHH children. Even when we control for language skills, we still find that DHH children are outperformed by their NH peers on empathic abilities that are more dependent on social learning such as cognitive empathy and prosocial motivation. This indicates that for fully-fledged empathic functioning sufficient language skills alone are not enough.

By observing how others interact we learn how to deal with our own and others emotions and to place them in a social context. This so-called incidental learning (i.e., learning by experience and with no educational intentions) is essential in order to develop empathic behavior [41]. Observing how a mother comforts her son after he lost his favorite football not only helps to understand how the boy feels (i.e., cognitive empathy) but also shows an adequate response (i.e., prosocial behavior). Since incidental learning often implies overhearing conversations between others with quick and snappy dialogues, missing the opportunity for this kind of learning will disadvantage DHH children.

For adequate cognitive empathy to develop a child needs to be able to recognize emotions in others [42]. Previously, lower levels of emotion recognition were reported in DHH toddlers [43] and school-aged children compared to NH peers [44]. This could explain the impaired level of cognitive empathy in the DHH group in our study. However, our study also indicates that DHH children are just as capable as their NH peers when it comes to recognizing emotions in others. It may be that with increasing age DHH children are able to catch up on this ability, and identification of emotions in others no longer seems to be the problem. It is the more complex interpretation of the whole emotion-evoking situation that causes confusion: why is my friend angry, what has happened?

The DHH population is often characterized by its heterogeneity (e.g., differences in degree of hearing loss, type and duration of hearing amplification, educational setting, mode of communication). In our study DHH children attending mainstream schools reported higher levels of cognitive empathy than DHH children in special schools for the Deaf and Hard of Hearing. Yet, we have to note that reasons for professionals to advise children to attend special education are diverse. Language skills and intelligence are factors influencing school placement in DHH children. Because these abilities can also influence empathic functioning, we considered them to be confounding factors. However, our study indicates that even if the levels of language skills and intelligence are equal, DHH children attending special education still have difficulties understanding others’ emotions. Despite these difficulties, DHH children in special education do not differ in their tendency to behave prosocial when compared to DHH children that attend mainstream education.

Children in special schools more often use sign language as their preferred mode of communication. In our study we found that children who use sign (supported) language showed less prosocial motivation. However, when comparing signers in special and mainstream education we found no differences in any of their empathic abilities. Previous studies reported differences in socio-emotional development between children with CIs and those wearing HAs in favor of the children wearing CIs. Our study indicates that when the child’s focus needs to shift to ‘the other’ instead of ‘the self’, these differences no longer appear and both groups show equal levels of empathy. Yet, these results have to be interpreted with caution as the groups used for these analyses were rather small.

It is important to note that the children in this study were born before the start of early detection and intervention programs in the Netherlands and Belgium. Therefore, these children were rehabilitated at a relatively late age (e.g., mean age at first hearing amplification 24.8 months, mean age at implantation 44,5 months). With the introduction of newborn-hearing screening programs, intervention and rehabilitation now preferably starts before the child is six months old [4]. As early intervention programs have been shown to improve speech and language skills, these improvements will hopefully lead to better communication skills, resulting in more effective incidental learning and higher empathic functioning. Future research is needed to define the impact of early intervention on these aspects of social-emotional development.

In conclusion, with this study we hope to have created awareness of the impaired empathic abilities of the DHH child. This will severely affect their social relationships, because there is a strong positive association between empathy and friendship quality in both NH and DHH children [45–48]. Lower empathic abilities influence a child’s social interaction, for example during play. For cooperative play with peers children need to share one another’s goals, desires, and beliefs [49]. Not being able to empathize with the other may result in less participation in play with others, causing isolation in the DHH child [50]. For their socio-emotional development DHH children benefit from achieving sufficient language skills. Yet, it takes more to obtain sufficient empathic abilities. If these abilities are to improve more attention could be paid to these issues in rehabilitation programs and family support. Professionals should create awareness concerning empathic abilities in the child’s surrounding. Parents and teachers can contribute to the development of empathic skills by actively involving the DHH child in emotion-evoking situations, or by talking about emotions more often. Future research should focus on the development of rehabilitation programs for DHH children that actively support the development of empathic abilities.

### Future studies

The psychometric properties of the empathy questionnaire were satisfying with good reliability in both DHH children and their NH peers. However, to assure that DHH children are as capable as hearing children in understanding the items well, further psychometric properties will be useful to examine. Item response theory models can shed further light on issues such as measurement invariance, which includes differential item functioning. Because of power issues we were not able to perform this type of analyses. Future studies with a larger cohort of DHH children are needed to address these issues. Regarding the design of this study, we have to point out that cross-sectional data were used, which prevents us from drawing conclusions about causality. Therefore, we started longitudinal data collection to confirm the assumptions made here.

## Supporting Information

S1. Dataset containing Deaf and Hard of Hearing participants and hearing controls. (SAV)
