# Correction: Low Empathy in Deaf and Hard of Hearing (Pre)Adolescents Compared to Normal Hearing Controls

**DOI:** 10.1371/journal.pone.0248546

**Published:** 2021-03-09

**Authors:** Anouk P. Netten, Carolien Rieffe, Stephanie C. P. M. Theunissen, Wim Soede, Evelien Dirks, Jeroen J. Briaire, Johan H. M. Frijns

## Notice of republication

An incorrect version of S1 Dataset was published in error. This article was republished on February 22, 2021, to correct for this error. Please download this article again to view the correct version.
